# Mismatch Negativity and Loudness Dependence of Auditory Evoked Potentials among Patients with Major Depressive Disorder, Bipolar II Disorder, and Bipolar I Disorder

**DOI:** 10.3390/brainsci10110789

**Published:** 2020-10-28

**Authors:** Yang Rae Kim, Young-Min Park

**Affiliations:** 1 Dr. Kim’s Hue Psychiatric Office, Bucheon 14548, Korea; proband@hanmail.net; 2 Department of Psychiatry, Inje Univeristy, Ilsan Paik Hospital, Goyang 10380, Korea

**Keywords:** mismatch negativity, LDAEP, major depressive disorder, bipolar II disorder, bipolar I disorder

## Abstract

Mismatch negativity (MMN) and loudness dependence of auditory evoked potentials (LDAEP), which are event-related potentials, have been investigated as biomarkers. MMN indicates the pre-attentive function, while LDAEP may be an index of central serotonergic activity. This study aimed to test whether MMN and LDAEP are useful biological markers for distinguishing patients with bipolar disorder (BD) and major depressive disorder (MDD), as well as the relationship between MMN and LDAEP. Fifty-five patients with major depressive episodes, aged 20 to 65 years, who had MDD (*n* = 17), BD type II (BIID) (*n* = 27), and BD type I (BID) (*n* = 11), were included based on medical records. Patients with MDD had a higher MMN amplitude than those with BID. In addition, the MMN amplitude in F4 positively correlated with the Korean version of mood disorder questionnaire scores (*r* = 0.37, *p* = 0.014), while the MMN amplitude in F3 correlated negatively with LDAEP (*r* = −0.30, *p* = 0.024). The odds ratios for the BID group and some variables were compared with those for the MDD group using multinomial logistic regression analysis. As a result, a significant reduction of MMN amplitude was found under BID diagnosis compared to MDD diagnosis (*p* = 0.015). This study supported the hypothesis that MMN amplitude differed according to MDD, BIID, and BID, and there was a relationship between MMN amplitude and LDAEP. These findings also suggested that BID patients had a reduced automatic and pre-attentive processing associated with serotonergic activity or N-methyl-D-aspartate receptor.

## 1. Introduction

Many studies using various methodologies have been conducted to distinguish bipolar disorder (BD) and major depressive disorder (MDD) [1]. However, no consistent results have been found yet. Recently, the possibility that cognitive impairment is one of the distinguishing factors between BD and MDD has emerged. Some studies have shown patients with BD in the euthymic state suffering from cognitive impairment, similar to those with schizophrenia [2,3]. This has contradicted old claims of a clear period of normalized functions in BD patients in inter-episodes, as opposed to those with schizophrenia. Thus, cognitive impairment may be a distinguishing trait marker for BD but not for MDD [4].

Mismatch negativity (MMN), an indicator of automatic change detection, is elicited by the temporo-frontal network [5] in response to a rarely presented deviant sound interspersed with frequently presented standard tones [6]. MMN research has been conducted on many neuropsychiatric conditions [7]. While the MMN amplitude is increased in healthy people with a higher visual working memory [8], the MMN amplitude reduction is associated with cognitive and functional deficits in patients with psychosis [9,10]. In particular, abnormally decreased MMN has often been reported in schizophrenia [11,12]. Despite the schizophrenia-related specificity of MMN, mounting evidence supports that MMN is a neurophysiological biomarker of intermediate effect in patients with BD [13]. MMN reduction is also associated with cortical thinning in temporal and frontal brain regions in patients with schizophrenia and BD [12]. Recently, two meta-analyses have revealed moderate effect sizes for MMN impairment in BD [14,15]. Additionally, MMN amplitudes have revealed negative correlations with functional outcomes in schizophrenia and neurocognitive functions in BD [12]. Thus, this finding suggests that cognitive impairment in BD and schizophrenia comes from common neurobiological disturbances [12]. On the contrary, MMN studies on MDD have shown mixed results. MMN abnormality is not a pathognomonic finding in patients with MDD. To summarize, MMN alterations can possibly be key distinguishing biomarkers between BD and MDD associated with the presence of cognitive impairment.

The loudness dependence of auditory evoked potentials (LDAEP), calculated as the slope of the linear regression using the peak-to-peak N1/P2 amplitudes elicited by five auditory stimuli intensities, is a biological marker of central serotonergic activity in neuropsychiatric diseases, despite gamma-aminobutyric acid and glutamate being also involved in generating auditory evoked potentials [16,17,18]. For example, a higher baseline LDAEP is associated with a better clinical response to the treatment with selective serotonin reuptake inhibitors and higher suicidality in patients with MDD [19,20]. In addition, LDAEP is significantly higher in patients with MDD than in those with BD or schizophrenia [21]. It suggests that people with BD and schizophrenia have a higher central serotonergic activity, the reason why clinicians prescribe serotonin antagonists (atypical antipsychotics) in those patients than to people with MDD. Furthermore, LDAEP has also been associated with childhood trauma [22]. Depressed patients with childhood trauma have higher LDAEP than those without childhood trauma. It suggests that childhood trauma may cause serotonin deficiency, affecting the development and clinical outcomes of depression. Thus, LDAEP can be an indicator of disease differentiation, treatment response, and association with childhood trauma.

However, to date, the relationship between MMN and LDAEP remains unknown. It has been recently found that acute tryptophan depletion (ATD) increases LDAEP and MMN amplitudes and shortens MMN latencies [23,24]. However, in another study, a selective serotonin reuptake inhibitor has been shown to increase MMN amplitude [25]. Given the contradictory results from these two studies, the question remains unanswered.

Thus, the aim of the current study was to test whether MMN and LDAEP are biological markers distinguishing BD and MDD patients. In addition, this is the first study to investigate the relationship between MMN and LDAEP.

## 2. Materials and Methods

### 2.1. Subjects and Study Design

In total, 55 patients with major depressive episodes, aged between 20 and 65 years, diagnosed either with MDD (*n* = 17), BD type II (BIID) (*n* = 27), or BD type I (BID) (*n* = 11), based on their medical records at the Ilsan Paik Hospital between 2016 and 2020, were included. The subjects with other major psychiatric disorders or personality disorders, as per the Diagnostic and Statistical Manual of Mental Disorders (DSM-5), were excluded. The subjects were divided into MDD, BIID, and BID groups according to the DSM-5 criteria. Clinical information, such as the results for Beck Depression Inventory (BDI), the Korean version of the Mood Disorder Questionnaire (K-MDQ), and the Korean version of Childhood Trauma Questionnaire (K-CTQ), and event-related potentials (ERPs), such as duration MMN and LDAEP, was obtained from the medical records. MMN and LDAEP were measured within 2 weeks after the initiation of medication. The K-CTQ is a self-report questionnaire that has five subscales of childhood abuse or neglect experience: physical abuse (PA), emotional abuse (EA), sexual abuse (SA), physical neglect (PN), and emotional neglect (EN). Each subscale consists of five items rated on a 5-point scale, from 1 (never true) to 5 (very often true).

The study protocol was approved by the ethics committee of Inje University Ilsan Paik Hospital, ethical approval code ISPAIK 2018-10-015.

### 2.2. MMN and Procedure

The patients were seated in a chair in front of a monitor (Mitsubishi, 22-inch CRT monitor) and asked to watch a silent film. The auditory stimuli were delivered via MDRD777 headphones (Sony, Tokyo, Japan) and consisted of sounds at 85 dB sound pressure level and 1000 Hz. Deviant tones lasting 100 ms were randomly presented, interspersed with standard tones lasting 50 ms (probabilities: 10% and 90%, respectively), which is called duration MMN. In total, 750 auditory stimuli were presented with an interstimulus interval of 500 ms. The experiment took about 10 min to complete. The stimuli were generated using E-Prime software (Psychology Software Tools).

Electroencephalography (EEG) recordings were synchronized to stimulus presentation onset by E-Prime. EEG was recorded using a NeuroScan SynAmps amplifier (Compumedics USA, El Paso, TX, USA) with 64 Ag-AgCl electrodes mounted on a Quik-Cap using an extended 10–20 placement scheme. The ground electrode was placed on the forehead, and the physically linked reference electrode was attached to both mastoids. The impedance was maintained below 5 kΩ. EEG data were recorded with a 0.1–100 Hz bandpass filter at a sampling rate of 1000 Hz, following the protocol described elsewhere [12]. The MMN wave was generated by subtracting the standard ERP wave from the deviant waves. MMN amplitude was measured as the mean voltage between 130 and 280 ms at three electrode sites (F3, Fz, and F4). If any remaining epochs contained significant artifacts (amplitude exceeding ± 75 μV), they were removed from further analysis. Only artifact-free epochs were averaged across trials and subjects for ERP analysis. The rejection rate was <1%. The number of epochs of deviant and standard stimuli used for the analysis did not significantly differ among patients with three groups.

### 2.3. LDAEP and Procedure

After the MMN measurement, N100 and P200 were measured. The auditory processing consisted of 1000 stimuli with an interstimulus interval of 500–900 ms. Tones at 1000 Hz and with a duration of 80 ms (with 10 ms rise and fall times) were generated by the same software at five intensities (60, 70, 80, 90, and 100 dB SPL) via the same headphones. EEG data were also recorded from 64 scalp sites, following a protocol described elsewhere [26]. The peak-to-peak N1/P2 amplitudes were measured for the five stimulus intensities, and the LDAEP was calculated as the slope of the linear regression curve.

### 2.4. Statistical Analysis

The subjects were divided into MDD, BIID, and BID groups according to their clinical diagnosis, based on DSM-5. The Kolmogorov–Smirnov test was used to check whether the clinical variables were normally distributed. Kruskal–Wallis test, Analysis of variance (ANOVA), Fisher’s exact test, and Spearman’s correlation test were used for group comparisons and relation strength. Differences between the three groups were calculated with subsequent post hoc analysis. Multinominal logistic regression was used to identify the association between MMN and clinical diagnosis. All tests were two-tailed, and the cutoff for significant group differences was *p* < 0.05. The statistical analysis was carried out using the SALT 2.5 software package (Istech Inc., Goyang, Korea).

## 3. Results

**Figure 1:**
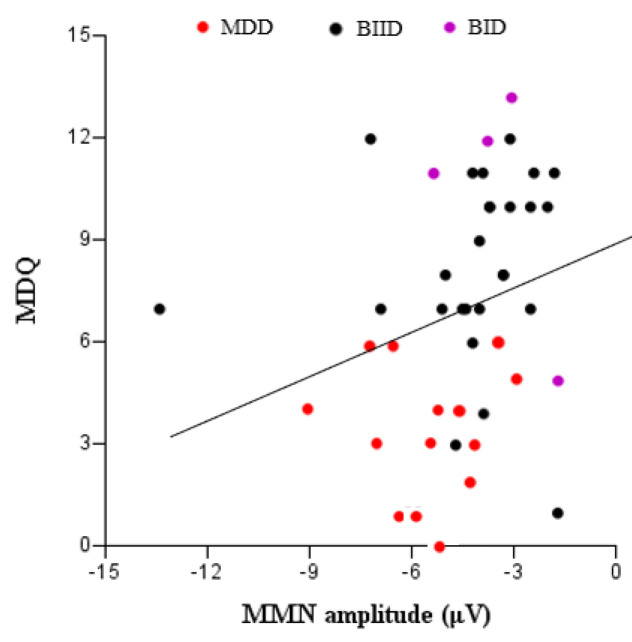
Correlation between K-MDQ (Korean version of the Mood Disorder Questionnaire) and the MMN (mismatch negativity) amplitude (F4) (*r* = −0.37, *p* = 0.014).

**Figure 2:**
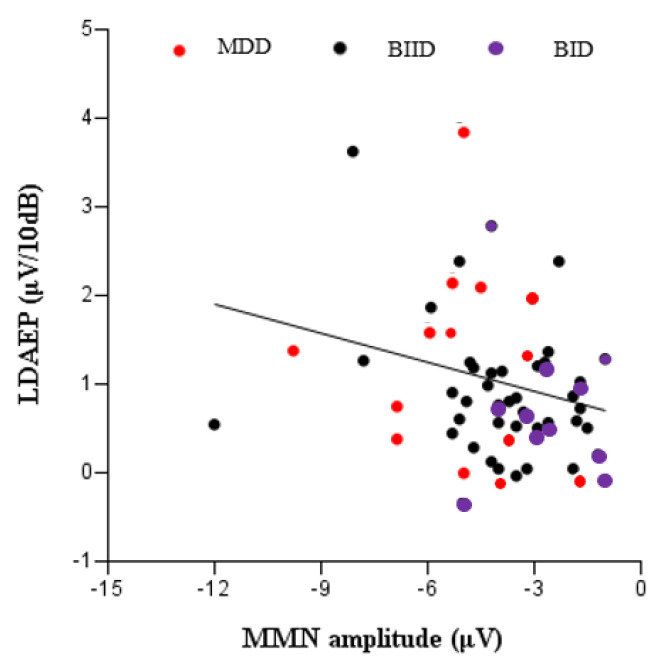
Correlation between LDAEP (loudness dependence of auditory evoked potentials) and MMN amplitude (F3) (*r* = −0.30, *p* = 0.024).

**Figure 3:**
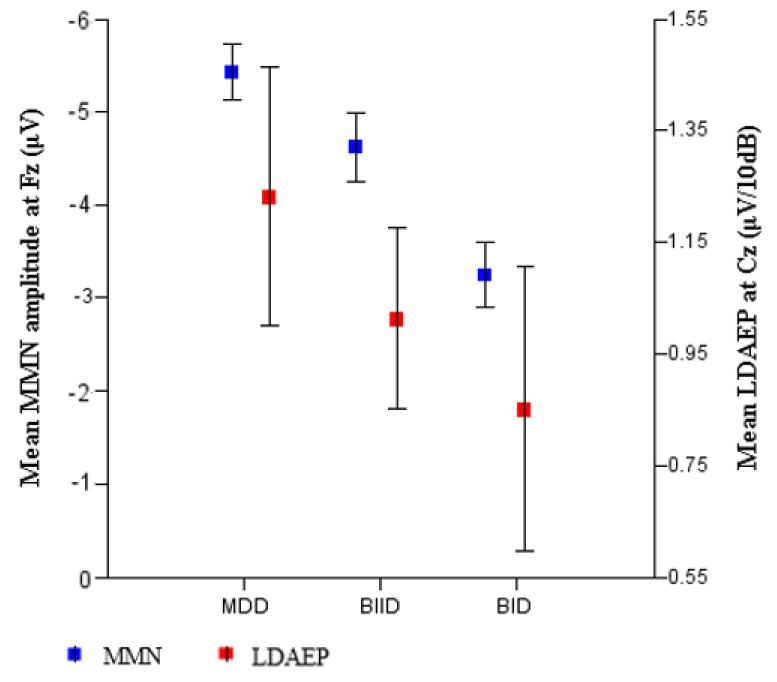
Mean MMN amplitude (Fz) (*p* = 0.026) and LDAEP (Cz) (*p* = 0.52) among patients with major depressive disorder, bipolar II disorder, and bipolar I disorder classified according to clinical diagnosis based on the DSM-5 (Diagnostic and Statistical Manual of Mental Disorders).

**Figure 4:**
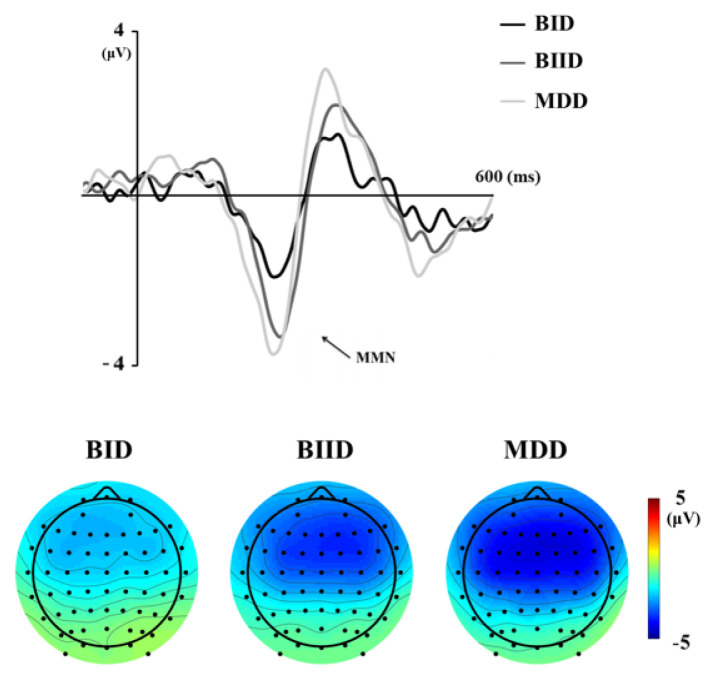
Mismatch negativity (MMN) waveforms and topographic MMN maps in the Fz electrode in the MDD (major depressive disorder), BIID (bipolar II disorder), and BID (bipolar I disorder) groups (Time frame ranges from 130 to 250 milliseconds).

**Figure 5:**
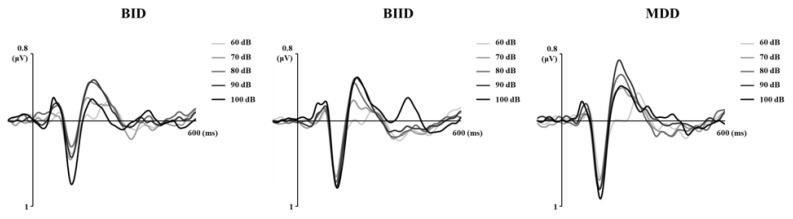

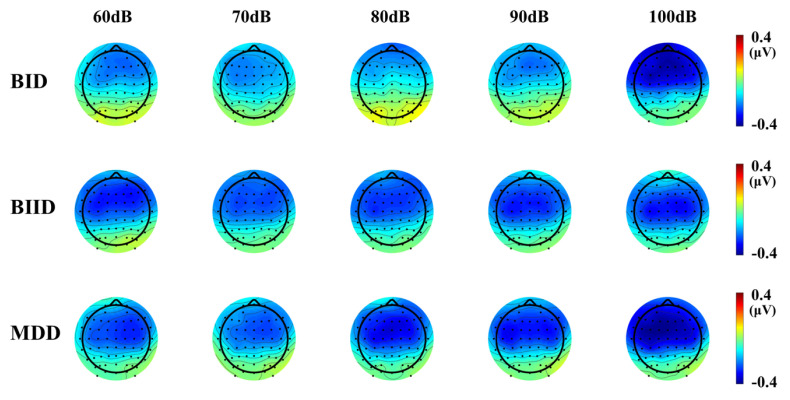
LDAEP (loudness dependence of auditory evoked potential) waveforms and LDAEP topographic maps in the Cz electrode in the MDD, BIID, and BID groups (Time frame ranges from 100 to 180 milliseconds).

We evaluated a total of 17 patients with MDD, 27 with BIID, and 11 with BID. They were divided into three groups according to their clinical diagnoses (Table 1). MMN amplitude in F3, Fz, and F4 differed among the three groups. A post hoc analysis revealed that MMN amplitude differed significantly between patients with MDD and BID (Table 1). In contrast, age, K-CTQ, BDI, and LDAEP did not differ among these three groups. In addition, the size of MMN amplitude negatively correlated with K-MDQ scores (F4) (*r* = 0.37, *p* = 0.014), while it positively correlated with LDAEP (*r* = −0.30, *p* = 0.024) (Figure 1 and Figure 2). However, these correlations were not found within each group. Figure 3, Figure 4 and Figure 5 show the MMN amplitude and LDAEP according to MDD, BIID, and BID. Table 2 lists the results of multinominal logistic regression. After adjusting for confounding factors (age, sex, and LDAEP), a significant MMN amplitude (F3) reduction was observed in BID patients compared to MDD patients (reference), although this association was not revealed in F4 and Fz. The odds ratio was 2.17 (*p* = 0.015). However, BIID diagnosis did not have a significant odds ratio for MMN amplitude compared to MDD diagnosis (reference). There was a significant relationship between F4 and Fz.

## 4. Discussion

This study compared clinical and ERPs variables among MDD, BIID, and BID groups. When the subjects were divided into these three groups according to the clinical diagnosis, K-MDQ and MMN in the frontal region differed among the groups. When the patients were divided into two groups according to BD, the group with BD had a higher K-MDQ, sexual abuse in K-CTQ, and a lower MMN amplitude in the frontal region than those with MDD. Thus, the main study finding was the difference in MMN amplitude according to the diagnosis. In addition, the size of the MMN amplitude negatively correlated with K-MDQ and positively with LDAEP.

Our findings revealed that the patients with BD had a reduced MMN amplitude in the frontal region, similar to patients with schizophrenia, compared to those with MDD. This result seemed to reflect some overlapping pathophysiological pathways in schizophrenia and BD at genetic and neurocognitive processing levels. Thus, MMN amplitude can be used as a distinguishing biomarker between BD and MDD. Previous studies have focused on patients with schizophrenia who consistently reported reduced MMN amplitudes to auditory stimuli compared to healthy controls [27]. However, reduced MMN amplitude has been observed in patients with BD or schizophrenia. In a previous study, patients with schizophrenia and BD have revealed reduced MMN amplitude in the frontal region compared to normal controls, with MMN amplitude not differing significantly between the two groups [12]. A recent study has also found substantial subsets of both schizophrenia and psychotic BD patients classified as neuropsychologically compromised and deteriorated in neuropsychological functioning and premorbid intellectual ability [28]. With the increasing number of MMN studies in BD, there is mounting evidence to support the intermediate effect of MMN as a neurophysiological biomarker in BD [13,29,30,31].

The current study revealed a lower MMN amplitude in patients with BD than in those with MDD. Thus, the MMN amplitude in patients with MDD seemed to be relatively intact compared to those with BD, although this study did not include healthy controls. However, studies on MMN abnormalities in patients with MDD have shown mixed results, with some reporting larger MMN amplitudes, while others have reported reduced values or no differences compared to healthy controls [32,33,34,35,36]. Some investigators have claimed MMN reduction to be a consequence of antidepressant treatment on cortical hyperexcitability [32], while others have considered these differences in MMN results due to the use of different deviant auditory stimuli for eliciting the MMN [32]. In addition, these mixed results could result from the heterogeneity of MDD [37].

MMN generation has been associated with the N-methyl-D-aspartate (NMDA) receptor [38]. A smaller MMN can predict psychotic experiences induced by NMDA receptor antagonists [39]. Additionally, ketamine has a significant influence on the latency and amplitude of MMN [40]. This is consistent with the hypothesis that the NMDA receptor hypofunction may mediate impaired auditory mismatch response in schizophrenia patients. Thus, MMN can be a useful marker of glutamatergic impairment [13].

LDAEP is significantly higher in patients with MDD than in those with BP or schizophrenia [21]; however, the current study did not reveal differences in LDAEP among MDD, BIID, and BID patients (Table 1). Nonetheless, as the MMN amplitude decreased, LDAEP also decreased significantly in the current study (Figure 2). This relationship between LDAEP and MMN amplitude seems to be associated with the NMDA receptor. Some investigators have also found that LDAEP is decreased by the NMDA antagonist MK-801 [41]. Collectively, it may be hypothesized that pre-attentive function reduces and central serotonergic activity or NMDA receptor impairment gradually increases with the frequency and intensity of psychotic symptomatology and cognitive impairment from MDD via BIID to BID.

A previous study has indicated that ATD increases MMN amplitudes to accommodate the duration and frequency changes in healthy subjects [23]. However, other studies have revealed ATD has either no effect on MMN amplitude, or it is reduced in healthy subjects [42,43]. Thus, the evidence for serotonergic modulation of MMN amplitude has not yet been confirmed. An alternative interpretation of the relationship between the MMN amplitude and LDAEP might be associated with the presence of psychotic symptoms. First-episode psychosis (FEP) of the schizophrenia-spectrum patients and FEP of the affective-spectrum patients are found to have similar MMN impairments as schizophrenia patients [44]. In addition, schizophrenia and psychotic BD have a lower LDAEP than normal controls and nonpsychotic BD patients [21,45].

In the current study, LDAEP did not differ among the MDD, BIID, and BID groups, suggesting that serotonergic activity did not differ among these groups. However, a previous study has revealed that LDAEP differs significantly among 123 patients with MDD and 37 patients with bipolar mania and bipolar depression [21]. This discrepancy could be due to the difference in the sample size, the state of bipolar illness, and the dosage of atypical antipsychotics (serotonin antagonists). Regarding the sex difference in LDAEP, some investigators have found a higher central serotonergic activity in female depressed patients than in male counterparts [46]. However, other studies, including the current one, have not found such differences [21,47]. Thus, this topic is still controversial.

This study has several limitations. First, the sample size was small; therefore, studies involving larger samples are needed in the future. Second, the current study did not include normal controls. However, it revealed the difference in MMN amplitude and the relationship between MMN and LDAEP according to clinical diagnosis. Third, we did not control the medication because the measurement of LDAEP and MMN was carried out, at the latest, within 2 weeks after the initiation of medication. Thus, future studies will need to evaluate MMN and LDAEP before the medication is initiated. Fourth, our MMN procedure was different from other studies in comparing short standards with long deviants. However, some studies have employed the same MMN procedure as ours [11,12,36]. In addition, sex distribution, duration of illness, and participants’ age distribution in each group could be potential limitations.

## 5. Conclusions

In conclusion, this study supported the hypothesis that the MMN amplitude differed according to MDD, BIID, and BID, and there was a relationship between the MMN amplitude and LDAEP.

## Figures and Tables

**Table 1:** Comparison of demographic and clinical variables among groups with major depressive disorder, bipolar I disorder, and bipolar II disorder classified according to clinical diagnosis based on the DSM-5.

Variable	MDD (*n* = 17)	BIID (*n* = 27)	BID (*n* = 11)	*F*	*p*	Post Hoc (Tukey)
^a^ Age, years	40.94 ± 17.81	33.00 ± 15.49	39.64 ± 10.44	3.67	0.16	
^b^ Sex, males/females	13/4	19/8	5/6	n/a	0.23	
^a^ LDAEP (µV/10dB), Cz	1.25 ± 0.96	1.02 ± 0.85	0.86 ± 0.85	1.32	0.52	
^c^ MMN latency, F3	227.35 ± 17.16	226.96 ± 20.55	243.00 ± 25.49	2.60	0.084	
^a^ MMN amplitude, F3	−4.92 ± 1.77	−4.16 ± 2.34	−2.87 ± 1.44	8.54	0.014 *	MDD > BID
^a^ MMN latency, Fz	227.65 ± 17.37	230.59 ± 18.16	241.18 ± 25.99	1.89	0.39	
^c^ MMN amplitude, Fz	−5.37 ± 1.59	−4.51 ± 2.41	−3.16 ± 1.60	3.93	0.026 *	MDD > BID
^c^MMN latency, F4	226.06 ± 17.88	229.37 ± 18.03	235.09 ± 24.64	0.72	0.49	
^a^ MMN amplitude, F4	−5.17 ± 1.57	−4.33 ± 2.31	−3.13 ± 1.54	9.03	0.011 *	MDD > BID
^c^ BDI	27.18 ± 14.38	27.28 ± 14.56	26.25 ± 13.53	0.008	0.992	
^a^ K-CTQ	47.07 ± 21.33	44.32 ± 17.83	30.25 ± 20.66	0.96	0.62	
^a^ Emotional abuse	10.07 ± 6.83	10.00 ± 5.10	6.67 ± 0.58	0.71	0.70	
^a^ Physical abuse	8.86 ± 5.67	9.38 ± 5.13	8.33 ± 3.06	0.24	0.89	
^a^ Sexual abuse	5.14 ± 0.54	6.17 ± 2.01	6.00 ± 1.73	4.28	0.12	
^c^ Emotional neglect	14.29 ± 6.68	12.38 ± 5.47	9.67 ± 3.22	0.95	0.40	
^a^ Physical neglect	8.71 ± 4.25	8.25 ± 3.14	9.67 ± 5.69	0.11	0.95	

* *p* < 0.05. Data are shown as mean standard deviation or percentage values. ^a^ Kruskal–Wallis test, ^b^ Fisher’s exact test, ^c^ ANOVA. Abbreviations: MDD = major depressive disorder; BIID = bipolar II disorder; BID = bipolar I disorder; LDAEP = loudness dependence of auditory evoked potentials; BDI = Beck Depression Inventory; K-CTQ = Korean version of the Childhood Trauma Questionnaire; MMN = mismatch negativity; DSM-5 = Diagnostic and Statistical Manual of Mental Disorders.

**Table 2:** Results of multinomial logistic regression analysis for the adjusted odds ratio of mismatch negativity (F3) in patients with bipolar I disorder compared to those with major depressive disorder.

Variables	Coefficient	SE	Wald	df	*p*-Value	Odd Ratio	95% Lower CI	95% Upper CI
Age	0.011	0.03	0.124	1	0.724	1.011	0.953	1.072
Gender	1.417	0.923	2.355	1	0.125	4.126	0.675	25.208
MMN amplitude	0.776	0.318	5.973	1	0.015*	2.173	1.166	4.049
LDAEP groups	0.031	0.898	0.001	1	0.973	1.031	0.177	5.999

* *p* < 0.05. Abbreviations: SE = standard error; df = degree of freedom; CI = confidence interval; MMN = mismatch negativity; LDAEP = loudness dependence of auditory evoked potentials.
